# SwAV-driven diagnostics: new perspectives on grading diabetic retinopathy from retinal photography

**DOI:** 10.3389/frobt.2024.1445565

**Published:** 2024-09-13

**Authors:** Md Nuho Ul Alam, Erfanul Hoque Bahadur, Abdul Kadar Muhammad Masum, Farzan M. Noori, Md Zia Uddin

**Affiliations:** ^1^ Department of Software Engineering, Daffodil International University, Dhaka, Bangladesh; ^2^ Department of Computer Science and Engineering, International Islamic University Chittagong, Chittagong, Bangladesh; ^3^ Department of Informatics, University of Oslo, Oslo, Norway; ^4^ Department of Sustainable Communication Technologies, Sintef Digital, Oslo, Norway

**Keywords:** diabetic retinopathy, contrasting clustering, SwAV, convolutional neural network, ensemble learning, transformer, early diagnosis

## Abstract

Diabetic Retinopathy (DR) is a serious eye condition that occurs due to high blood sugar levels in patients with Diabetes Mellitus. If left untreated, DR can potentially result in blindness. Using automated neural network-based methods to grade DR shows potential for early detection. However, the uneven and non-quadrilateral forms of DR lesions provide difficulties for traditional Convolutional Neural Network (CNN)-based architectures. To address this challenge and explore a novel algorithm architecture, this work delves into the usage of contrasting cluster assignments in retinal fundus images with the Swapping Assignments between multiple Views (SwAV) algorithm for DR grading. An ablation study was made where SwAV outperformed other CNN and Transformer-based models, independently and in ensemble configurations with an accuracy of 87.00% despite having fewer parameters and layers. The proposed approach outperforms existing state-of-the-art models regarding classification metrics, complexity, and prediction time. The findings offer great potential for medical practitioners, allowing for more accurate diagnosis of DR and earlier treatments to avoid visual loss.

## 1 Introduction

Diabetic Retinopathy (DR) is a progressive retinal condition that is considered the most prevalent and common consequence of diabetes mellitus Nathan (1993). A comprehensive review by Teo et al. (2021) highlighted that DR stands as a leading contributor to avoidable vision impairment among the worldwide working-age population. The study also reported that in 2020, the global prevalence of this illness topped 103.12 million. According to predictions by that study, the total will rise to around 160.50 million by 2045. In persons aged 50 and older, DR was the seventh largest reason for blindness and moderate to severe visual impairment. It is a slowly developing hidden chronic disease that often occurs in people with diabetes. DR does not show clear early signs; as it worsens, complete blindness is the likely outcome.

**FIGURE 1:**
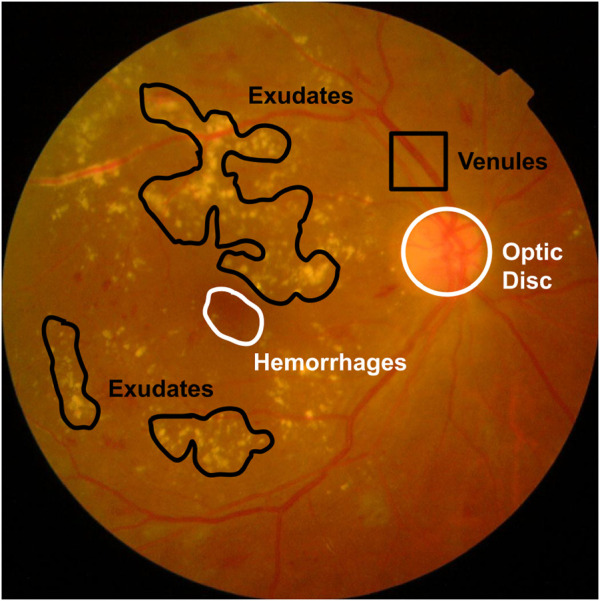
Various lesions from a fundus image.

Regular screening helps catch DR early, preventing further damage with the right medication. High-resolution fundus images are used to spot tiny lesions and assess their severity. DR comes in two main forms: Proliferative DR (PDR) and Non-proliferative DR (NPDR). NPDR comprises four levels of severity—severity level 0 to 3 (Fong et al., 2004). Figure 1 displays typical DR symptoms. A microaneurysm (MA) manifests as a small, dark red dot-like lesion observed at the terminal of a blood vessel (Manjiri et al., 2015). Hypertension and blockage of the retinal veins have the potential to result in retinal hemorrhage (HM), which is another complication associated with DR (Kanukollu and Ahmad, 2023). Occasionally, small retinal hemorrhages (HM) may bear resemblance to microaneurysms (MA) (Soriano and Aguirre, 2016). Exudates, which include lipids and protein residues, filter out wounded capillaries. In its latter stages, DR is difficult to treat. The Mild NPDR shows only a few microaneurysms. In contrast, moderate NPDR patients experience many MAs, hemorrhages, and venous beading, which compromises their ability to move blood to the retina. Severe DR is characterized by above 20 intraretinal hemorrhages per quadrant, at least two quadrants with noticeable venous beading, and significant intraretinal microvascular abnormalities (IRMA) in at least one quadrant. During the PDR stage, new blood vessels (neovascularization) emerge with vitreous or pre-retinal hemorrhages (Shukla and Tripathy, 2023). Fundus imaging is utilized for the diagnosis of DR. Ophthalmologists evaluate fundus images for visible lesions, assess a DR level, and recommend suitable treatment options.

**FIGURE 2:**
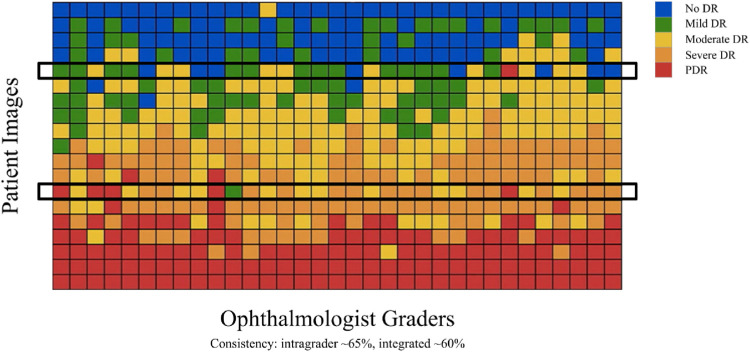
Inconsistency among ophthalmologists Google (2017).

Expert ophthalmologists struggle to produce reliable diagnoses for fundus images due to overlapping borders and minor lesions, making the process time-consuming. Therefore, the scientific community recognizes the urgent necessity for a computer-aided system in DR grading. However, developing such systems requires annotated and labeled datasets from expert ophthalmologists, which adds to the challenge given the variability in doctors’ assessments, as illustrated in Figure 2 taken from Google (2017) where each row represents patient retinal fundus images and each column is DR gradings by US-board certified ophthalmologists. This variability includes differences in severity level categorizations, with one doctor labeling an eye as severity level-3 (Severe DR) while another may classify it as level-1 (Mild DR), and some even assigning a severity rating of 4 (PDR). Therefore, relying solely on models trained with noisy annotated DR data by ophthalmologists is not feasible for real-life applications. Acquiring high-quality datasets with accurately labeled images is crucial in deep learning applications, particularly in healthcare, where compromising quality is not an option. However, obtaining properly labeled DR images remains challenging, highlighting the need for an automated DR grading system that does not depend on annotated or labeled datasets.

This study introduces a novel approach for grading Diabetic Retinopathy (DR) using the self-supervised Contrasting Cluster Assignment algorithm SwAV (Caron et al., 2020) on the benchmark APTOS 2019 retinal fundus image dataset (Asia Pacific Tele-Ophthalmology Society, 2019). First, the dataset undergoes some preprocessing steps and afterward is fed to train a contrastive learning-based model SwAV. Generally, contrastive learning is a useful unsupervised method of learning visual elements. Rather than predicting a label for a picture, contrastive approaches train convolutional networks by differentiating between images. The method compares multiple augmented views of original photos to a different image, such as a monochromatic and color photo of a retinal fundus image, and a color drawing of image from different domain. It may thus conclude that, despite their apparent differences, the third lacks semantic information, but the first two do provide somewhat similar information. By using the information that visually separates pictures, contrastive learning may identify semantics in them. In practice, contrastive learning utilizes two alternative transformations of the mentioned fundus image and pushes both closer together than other image changes. This lets the model understand that the concept of a fundus image grade is resilient to image alterations that change, for example, its color. This strategy works well, but it needs the system to change the same image several times and compare each altered image independently. This is an incredibly computationally hard process. SwAV does not need explicit comparisons between each image pair. First, characteristics from clipped regions of two photos are calculated and assigned to an image cluster. These are distinct assignments that are not going to match. While the color version of the fundus image may match a cluster with various fundus images, the monochromatic version might match an image cluster with certain retinal fundus images. The system restricts the alignment of the two cluster assignments over time, ultimately resulting in the discovery that all retinal fundus images convey identical information. This is accomplished by comparing the cluster assignments or anticipating the cluster of one type of image over the other.

The suggested method based on SwAV with ResNet50 inside outperforms the most state-of-the-art established techniques such as CNN and Transformer-based model networks (Table 4). Because the technique is quick and efficient, it may be used in real-life healthcare applications. An ablation study was performed to discover the performance difference between SwAV and the combination of different CNN with transformer-based supervised model networks.

Using just retinal fundus images, the system determines the possibility of Diabetic Retinopathy (DR) and can inform a patient of its severity. This motivates patients to seek clinical testing procedures based on the severity of their DR, decreasing dependency on costly routine medical visits until the system indicates an early diagnosis. Furthermore, the necessity for people to rely on obvious signs is eliminated. The system’s early diagnosis can assist avoid or delay the onset of DR-related problems. Regular screening is critical for avoiding DR-induced blindness. As the only technique that uses computer-based fundus image grading, it represents a novel and promising approach in medical science.

The novel contributions of this work are summarized as follows:

• Introduced a novel online clustering-based self-supervised approach, SwAV, for multiclass DR grading.

• Addressed the limitations of traditional contrastive learning methods for DR grading by clustering data to avoid direct pairwise comparisons of lesions and ensuring consistency between augmented views of the same image.

• The proposed method improves the handling of irregular and complex lesion features in DR images, where conventional CNNs and transformer-based models often struggle.

• The proposed approach reduces the dependency on large amounts of labeled DR fundus image data, making it more practical in sectors where obtaining annotated data is challenging.

• The proposed model is simpler and more efficient than state-of-the-art models, with only 16 layers and 23.5 million parameters. This makes it faster to train and suitable for real-time applications, as it can classify fundus images in under 2 microseconds - a significant advantage for practical medical use.

• Compared to other state-of-the-art approaches, the proposed model significantly improves sensitivity, which is crucial for medical applications, ensuring better identification of affected patients and reducing the risk of misclassification.

## 2 Literature review

Noninsulin-dependent diabetes (NIDDM) patients often avoid routine eye screening likely due to time restrictions, absence of symptoms, and limited chances of meeting specialists (Chou et al., 2014). Thus, the likelihood of acquiring DR increases. One attempt to address this is the use of techniques based on AI for disease identification and diagnosis. Several computer-assisted solutions have been suggested for Diabetic Retinopathy severity grading. Ophthalmologists assess fundus image severity by screening lesions and treating them accordingly. Lesion segmentation methods were developed to help ophthalmologists accurately diagnose small lesions. Image processing methods were often utilized to segment fundus image lesions. Image processing techniques were employed by Mumtaz et al. (2018) to automatically identify hemorrhage, a common sign of retinal diseases among diabetes patients. Akram et al. (2014) found microaneurysms in tiny patches recovered from fundus images and utilized PCA to reduce dimensionality. A fuzzy C-means (FCM) image processing was employed by Rahim et al. (2016) to automatically diagnose maculopathy and Diabetic Retinopathy. A four-step lesion identification approach was created by Kar and Maity (2017) which involves extracting vessels and removing the optic disc, pre-processing, detecting potential lesions, and post-processing. To distinguish black lesions from poorly light retinal backgrounds, curvelet-based edge improvement was used, while a well-designed wideband bandpass filter increased the contrast between the background and the luminous lesions. Simultaneously, the mutual information of the highest matched filter response and the largest Laplacian of Gaussian response was maximized. Post-processing was performed using a morphological approach to remove erroneously detected pixels. Using image processing, Umapathy et al. (2019) extracted texture features which were classified using the Decision Tree (DT) model. The authors used transfer learning as their second strategy. Using image processing to extract complex features resulted in lower accuracy. Therefore, for lesion segmentation, deep learning architectures were introduced. Chudzik et al. (2018) used a CNN architecture to segment microaneurysms. Yu et al. (2017) also used CNN for pixel-base exudate detection. A semi-supervised CNN model was proposed by Gondal et al. (2017) for designating areas in retinal images. They reported high classification and sensitivity scores. Shenavarmasouleh and Arabnia (2021) introduced the Mask-RCNN model for segmenting tiny lesions, including MA and exudates. By reusing the weights of pre-trained ResNet101 using transfer learning (TL), the authors were able to achieve a 45% mAP score. Apart from segmentation, one popular technique for DR grading is image classification. Images are divided into classes based on distinct properties at the image level.

Several research used classic ML algorithms, including DT, SVM, RF, LR, and GNB. To use classical ML models for Diabetic Retinopathy grading, image processing techniques were used in feature extraction and then used to train models. Lachure et al. (2015) used morphology-based image processing methods to distinguish between MAs and exudates, including erosion, dilatation, opening, and closure. The characteristics were supplied into SVM and KNN classifiers to grade the fundus images. Asha and Karpagavalli (2015) used machine learning approaches to detect retinal exudates. The fuzzy C means method was utilized to segment the fundus images, and the Luv color space was utilized to identify the exudate properties. The classifiers used Native Bayes, Multi-Layer Perceptron (MLP), and Extreme Learning Machine (ELM). The best scores were obtained via ELM. (Honnungar et al. (2016) investigated machine learning algorithms for automatically classifying DR from retina images. Their suggested approach included classifying the images into distinct DR levels using a multi-class classifier (logistic regression, SVM, and RF), extracting features using the bag of visual words model, and preprocessing the images using Contrast Limited Adaptive Histogram Equalization (CLAHE). CLAHE was also used by Raman et al. (2016) to enhance images. In their work, morphological operation for blood vessel segmentation, areas expanding for exudates segmentation, Sobel operator and contour with circular hough transformation for optic disk segmentation, and a mixture model for microaneurysm segmentation come next. Finally, ANN was employed for classification. Image processing techniques were also used by Carrera et al. (2017) to extract characteristics from microaneurysms, blood vessels, and hard exudates. Later, the features were applied to a Support Vector Machine (SVM) classifier. They attained 95% sensitivity and 94% accuracy. SK and P (2017) developed an ensemble classifier known as ML-BEC, utilizing machine learning bagging techniques, which extracts features using t-distribution Stochastic Neighbor Embedding (t-SNE). A two-tier DR grading system was presented by Ramani and Lakshmi (2017). In their ensemble method, misclassified instances were eliminated and deployed to second-level ensemble classifiers utilizing J48 Graft Trees. Best First Trees (BFTs) were utilized in this process. Chetoui et al. (2018) used Long-tailed Prompt Tuning and Local Energy-based Shape Histogram (LESH) to identify textural properties. SVM was utilized for classification, along with several kernel functions. A histogram binning technique was employed to represent the features. The study found that LESH outperformed SVM using an RBF kernel, achieving a 90% accuracy rate. Few machine learning algorithms were used by Ali et al. (2020) for segmenting and categorizing DR images. They recommended clustering as a new paradigm for regional growth. For texture analysis, four character kinds were used: run-length matrix (RLM), co-occurrence matrix (COM), wavelet (W) and histogram (H). The authors improved classification accuracy by creating hybrid-feature datasets through the use of data fusion. By employing Fisher, the likelihood of error plus average correlation, mutual information, as well as correlation-based feature selection, they successfully identified the top 13 features. Finally, they utilized five classifiers: SLg (simple logistic), MLP (multilayer perceptron), Lg (logistic), and SMO (sequential minimal optimization). A multipath convolutional neural network (M-CNN) was created by Gayathri et al. (2021) to extract both local and global features from fundus images. The final DR classification was predicted using the J48, RF, and SVM models. When the J48 classifier was used, the M-CNN network produced the best results. A hybrid inductive machine learning algorithm (HIMLA) was proposed by Mahmoud et al. (2023) to automate DR detection. After the normalization of fundus images, segmentation of Blood vessels was done using a convolutional encoder-decoder. The approach of multiple-instance learning was applied for both feature extraction and classification. An ensemble learning technique was tested by Reddy et al. (2020) using Logistic Regression, KNN, Decision Tree, Random Forest, and Adaboost. The grid search method was employed by the authors to adjust the hyperparameters. An ensemble method combining SVM for accurate and faster prediction, Neural Network for improved precision, and Random Forest for persistent training was proposed by Odeh et al. (2021). The wrapper subset and information gain attribute evaluation algorithms were used by the authors to choose features.

Traditional machine learning requires the extraction of complicated characteristics as a first step. Manual feature extraction with image processing may not capture all complex characteristics required for proper categorization. Deep learning (DL) is currently widely employed in imaging for many purposes. DL models successfully identified DRs by extracting complicated features using convolutional layers. Islam et al. (2018) employed a CNN model featuring a kernel size of 4 × 4, coupled with augmentation and preprocessing strategies for detecting DR. By utilizing dropout and L2 regularizer, the authors were able to prevent overfitting and attain an 85% kappa score, sensitivity of 98%, and specificity of 94%. A multitasking DNN architecture was presented by Zhou et al. (2018) for DR classification task. The authors used a multitasking strategy to predict labels using classification and regression, resulting in an 84% kappa score due to the interdependence of the DR phases. Zeng et al. (2019) presented a Siamese-like architecture for DR detection. The model was learned using a transfer learning technique on binocular fundus pictures. Zhao et al. (2019) also proposed BiRA-Net, which was based on attention layers. Islam et al. (2020) developed a transfer learning model using VGG16 architecture. They used color preprocessing techniques. To address overfitting issues, they employed stratified K-fold cross-validation. Similarly, Samanta et al. (2020) also developed a transfer learning model using DenseNet architecture for a smaller Kaggle dataset. The authors achieved 0.8836 kappa score on validation. A multi-tasking DNN architecture was created by Majumder and Kehtarnavaz (2021) that detects five grades of DR. For interdependency, their architecture consists of a classification and a regression model. For the EyePACS dataset, they attained an 88% kappa score, and for the APTOS dataset, they attained 90%. Furthermore, Chen et al. (2020) presented an integrated shallow network. Gulshan et al. (2016) suggested an Inception-v3 network trained on 0.13 million retinal fundus images and assessed by 54 board-certified ophthalmologists in the United States. The model was evaluated on two distinct datasets categorized by seven board-certified ophthalmologists from the United States, and it achieved an AUC of 0.97–0.99 for detecting referable DR. Gulshan et al. (2019) confirmed the DR grading system’s performance at two Indian sites compared to manual grading. 82.18% classification accuracy attained by Gangwar and Ravi (2021) using an Inception-ResNet-v2 model on the Messidor-1 dataset. Their methodology was also tested on the APTOS 2019 dataset, achieving a classification accuracy of 72.33%. Kassani et al. (2019) created an Xception architecture with CNN layers and achieved 83.09% accuracy on the APTOS 2019 dataset, outperforming previous CNN-based pre-trained models. Bodapati et al. (2020) trained a DNN with blended multimodal deep features, achieving around 81% accuracy on the APTOS 2019 dataset. The characteristics were retrieved via multiple pre-trained CNN architectures. Bodapati et al. (2021a) trained a composite gated attention DNN that performed somewhat better than previously, with an accuracy of 82.5%. Adem (2018) demonstrated that, from a retinal image, segmenting the optic disc area before training CNN yields superior results than CNN-only techniques.

CNNs are clearly the most favored in image processing and applications involving computer vision. Conventional methods of convolutional neural networks and transformers consider images as grid or sequence structures, that may not be suitable for collecting irregular and complicated objects. While several models have been established, there is always scope for improvement, notably in multiclass categorization. Although ML architectures used in the study were less complicated than previous DL models, their classification performance fell short. Researchers employed several transfer learning (TL) models to improve classification performance and overcome drawbacks. TL models can be time-consuming to train due to their large number of parameters and layers. This paper provides a framework that balances ML and DL models to improve the performance of classification metrics while minimizing the total number of layers and parameters, thus saving training time. Furthermore, training convolutional networks often needs a substantial quantity of labeled data, limiting their usefulness in sectors where annotations are difficult to get. A key gap in previous research is the absence of a self-supervised contrastive learning strategy in DR grading. Recent advancements in self-supervised training methods, especially contrastive approaches like PIRL (Misra and Maaten, 2020) and MoCo (He et al., 2020), have made them a viable alternative to traditional supervised training. However, these systems have lagged in terms of performance and are substantially slower to train, frequently requiring 100 times the processing power of their supervised equivalents. To address this shortcoming, this study investigated SwAV for the DR grading task, as it uses contrastive learning in a significantly more efficient and effective manner.

## 3 Datasets description

**FIGURE 3:**
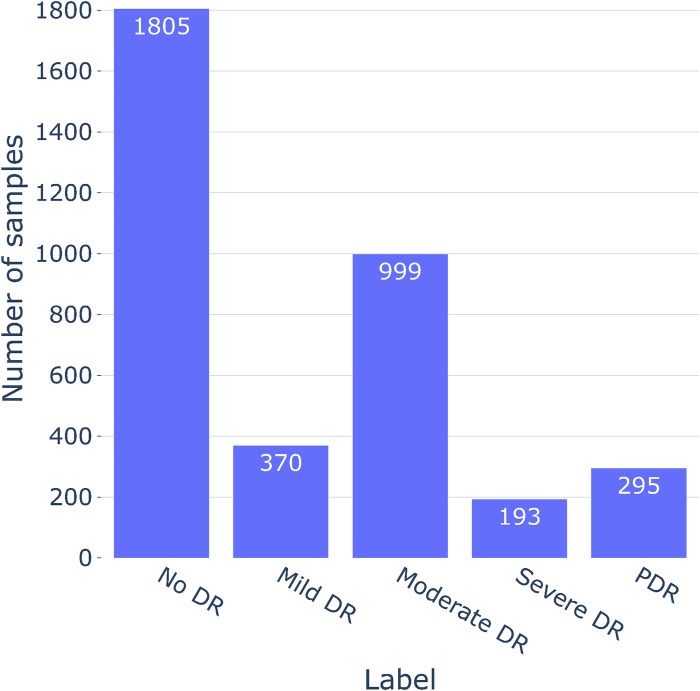
The count of fundus images within each class present in the APTOS 2019 dataset.

**FIGURE 4:**
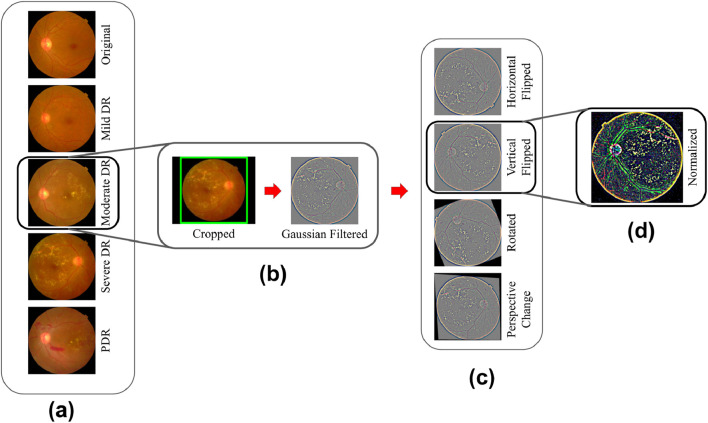
**(A)** APTOS 2019 dataset samples from each class; **(B,C)** Preprocessing step; **(C)** Augmentations; **(D)** Gaussian Filtration step.

The study utilized the APTOS 2019 (Asia Pacific Tele-Ophthalmology Society, 2019) dataset to evaluate the severity of DR. The dataset was contributed by technicians from the Aravind Eye Hospital, who utilized fundus photography to capture images from patients residing in rural areas. A wide variety of imaging situations are represented in the photographs. The images underwent grading for DR severity on a scale of 0–4 by a clinician. As the Aptos 2019 dataset was sourced from a Kaggle competition, the test photos associated with it were kept confidential. Consequently, all 3,662 training fundus photos from the competition were utilized for both model training and testing purposes. For additional details on the distribution of fundus images and sample representations for each severity class, refer to Figures 3, 4A, respectively.

## 4 Methodology

### 4.1 Pre-processing fundus images

Out of the 3,662 fundus images, 2,934 (80%) were utilized during the training phase. Within the training split, the counts of images for No DR, Mild DR, Moderate DR, Severe DR, and PDR were 1,445, 297, 800, 155, and 237, respectively. The remaining 728 (20%) images were used for testing. In the testing set, there were 360 images for No DR, 73 for Mild DR, 199 for Moderate DR, 38 for Severe DR, and 58 for PDR. To mitigate model overfitting and prevent the learning of erroneous patterns, fundus images underwent resizing and cropping of uninformative sections. Images exceeding 1,024 pixels were adjusted into a width value of 1,024 pixels during the adjustment process. To preserve the original object composition required for lesion detection, the aspect ratio was maintained during that process. Notably, the images had irregular black gaps surrounding the retina, potentially influencing the models on learning unimportant features. To address this issue, the spherical z-space was cropped. Cropping was done based on the fundus images radius value (see Figure 4B—Cropped).

An issue of uneven brightness was observed in the dataset images, leading to some images appearing excessively dark and hindering lesion visibility. This problem was compounded by the diverse imaging settings under which the images were captured. A Gaussian filter was used to balance brightness, improve model performance and facilitate the differentiation of lesions such as abnormal blood vessel development, aneurysms, cotton wool patches, hard exudates, and hemorrhages. An example of Gaussian filtered image is shown in Figure 4B.

Augmentations (as shown in Figure 4C—Horizontal Flipped, Vertical Flipped, Rotated, Perspective Change) were used to strengthen models against noisy data and enhance generalization by diversifying training samples. Overfitting occurs when a model becomes excessively specialized in recognizing patterns from noisy data. This leads to high variance and limited generalizability to new samples. In order to prevent the model from memorizing specific details and to improve its flexibility across a range of settings, image augmentation introduces diversity into the training dataset. Each image per epoch either got none at all or any among the four augmentations (Horizontal Flipped, Vertical Flipped, Rotated, Perspective Change) at random.

Upon loading an RGB image into memory, the values of its pixels vary from 0 to 255, with each channel represented as an integer of 8-bit. However, deep learning models often favor handling floating-point values in a narrow range. To accommodate this preference, the images underwent a Z-score normalization process. This technique aims to decrease skewness in the data distribution and enhance the stability of the model during training. The z-score normalization technique was applied and proved effective since the dataset did not have notable outliers that needed to be trimmed. For this normalization process, the mean and standard deviation were computed for each channel (refer to Table 1). These statistical values were then used in the normalization process to center the data around 0 and bring it within the range of −10 to 10, which contributed to quicker convergence. Figure 4D showcases a retinal fundus image post-normalization.

**TABLE 1:** Each channel Standard deviation (σ) and Mean (μ) values during normalization.

Channel	Standard deviation (σ)	Mean (μ)
Red	0.10	0.50
Green	0.08	0.50
Blue	0.04	0.50

**FIGURE 5:**
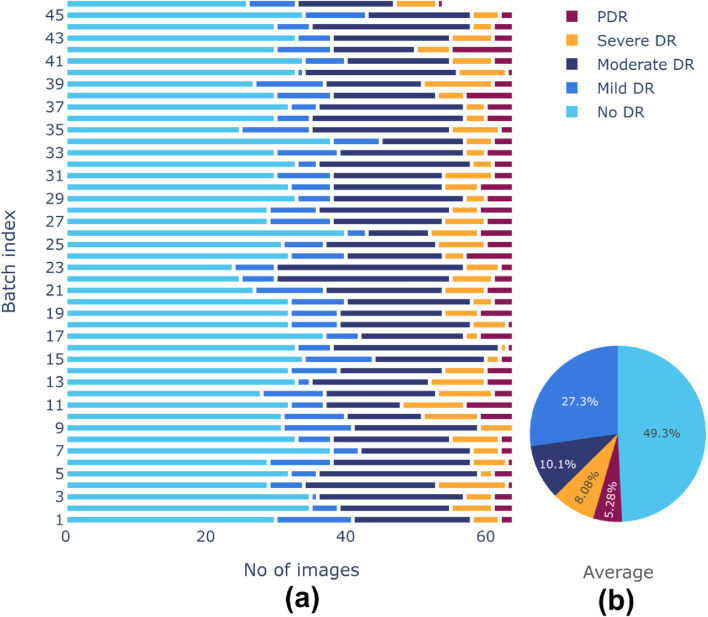
**(A)** Class distributions and **(B)** Average representation of classes before sampling of training dataset.

**FIGURE 6:**
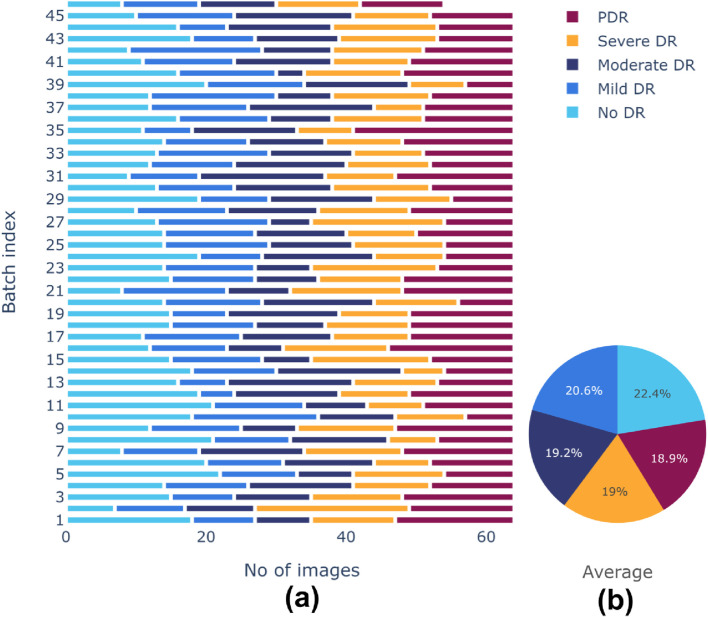
**(A)** Class distributions and **(B)** Average representation of classes after sampling of training dataset.

There is a class imbalance in the Aptos 2019 dataset, with notable variations in the number of samples between classes. This could lead to a biased model during training. A random-oversampling strategy was utilized to obtain equal class distributions across training batches. In order to balance the dataset, the oversampling procedure randomly picks pre-existing original samples from the minority classes and duplicates them batch by batch during the training process. To avoid potential issues of overfitting due to the repeated presence of identical samples, random oversampling was combined with data augmentation techniques elaborated before. These techniques introduced variability in the duplicated samples, helping to maintain dataset diversity and enhancing the generalization ability of the model. After oversampling, training batches produced more balanced class distributions. Before and after effects of sampling are shown in Figures 5, 6, respectively. The pie chart representation in Figure 6B demonstrates that, upon sampling, the average representation was about identical for all batches. This consistency made sure that throughout the training phase, the model acquired a sufficient proportion of image data from every class.

### 4.2 Contrasting cluster assignment with SwAV

Contrastive learning approaches have made unsupervised image representations more effective than supervised pretraining. These contrastive approaches are often used online and need a high number of direct pairwise feature comparisons, making them computationally tricky. SwAV (Caron et al., 2020) is an online algorithm that uses contrastive approaches without having to calculate pairwise comparisons. This approach clusters data and ensures consistency between augmentations or views of the same image, instead of explicitly comparing characteristics like contrastive learning. SwAV employs a “swapped” prediction method for predicting a view’s code based on its representation in another view. This approach is adaptable to both sizable and modest batches during training and possesses the capacity to handle extensive datasets effortlessly. In contrast to earlier contrastive techniques, it exhibits superior memory efficiency, as it operates without the need for extensive memory banks or specialized momentum networks.

One of the many objectives of this study is to research the visual characteristics of DR images in an online method without supervision. This work employed the online clustering-based self-supervised approach SwAV (Caron et al., 2020). According to Asano et al. (2019) and Caron et al. (2018), typical clustering-based deep neural network algorithms follow a pattern of alternating between an offline cluster assignment phase, and a training phase. In the cluster assignment phase, all image features are clustered. And “codes” or cluster assignments are predicted for distinct image augmentations in the training phase. However, these approaches are unsuitable for online learning due to the requirement for multiple iterations through the dataset to compute the necessary image features for clustering.

Here, a distinct approach is applied, enforcing consistency amongst codes derived from the additional augmented views of the actual retinal image. The approach draws inspiration from the work of Wu et al. (2018), where they demonstrated contrastive instance learning, prioritizing consistent mappings between various views of the original image instead of treating codes as targets. Essentially, the technique can be explained as contrasting numerous image views by differentiating the cluster assignments rather than the features.

**FIGURE 7:**
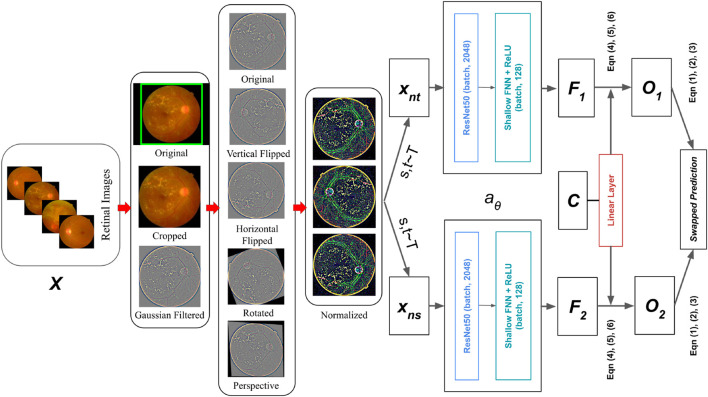
Retinal Fundus image classification architecture using Contrastive Clustering based SwAV online algorithm.

Specifically, in Figure 7, The codes are derived from a single augmented perspective of the original retinal fundus image, and then inferred from various other augmented representations of the identical retinal fundus image. Let there are two image features $f_{t}$ and $f_{s}$ from distinct augmented views of the identical image. The augmented views corresponding codes $o_{t}$ and $o_{s}$ get determined by aligning them with K prototypes set, denoted as $\left\{c_{1},\ldots ,c_{K}\right\}$ . Through the use of the following loss function (Equation 1), a “swapped” prediction task is formulated.

$$L\left(f_{t},f_{s}\right)=l\left(f_{t},o_{s}\right)+l\left(f_{s},o_{t}\right),$$ (1)

where, the function l(f,o) assesses the match between codes o and features f . Intuitively, this technique compares the features $f_{s}$ and $f_{t}$ via intermediate codes $o_{s}$ and $o_{t}$ . The other feature can predict the code if both features contain the same information. The information between two features can be deemed identical if they have the lowest loss compared to all other features. For an augmented view of an image, the set of B feature vectors denoted by $F=\left[f_{1},\ldots ,f_{B}\right]$ and the corresponding codes $O=\left[o_{1},\ldots ,o_{B}\right]$ . As $f_{t}$ and $f_{s}$ are two image features from two different augmented views of the same image, the feature $f_{t}$ belongs to the set of feature vectors $F_{1}=\left[f_{1t},f_{2t},\ldots ,f_{Bt}\right]$ for one view, while the feature $f_{s}$ belongs to the set $F_{2}=\left[f_{1s},f_{2s},\ldots ,f_{Bs}\right]$ for the other view. Similarly, the code $o_{t}$ belongs to the set of codes $O_{1}=\left[o_{1t},o_{2t},\ldots ,o_{Bt}\right]$ for one view, and the code $o_{s}$ belongs to the set $O_{2}=\left[o_{1s},o_{2s},\ldots ,o_{Bs}\right]$ for the other view.

Let, the dataset images are $X=\left[x_{1},x_{2},x_{3},\ldots ,x_{N}\right]$ and n=1,2,3,…,N . A transformation t or s is chosen from a set of transformations for images T to convert the retinal fundus picture $x_{n}$ to $x_{nt}$ or $x_{ns}$ . $x_{nt}$ and $x_{ns}$ are two different augmented views of an image. This approach generates multiple views of a batch of retinal fundus images by employing multi-crop and other augmentation techniques such as random grayscaling, flipping, and color distortion. Initially, these views are processed by a ResNet50 backbone to extract the embedding vector (the final Global Average Pooling layer output). Through a non-linear mapping $a_{\theta }$ , the augmented view $x_{nt}$ is mapped to a vector representation. The non-linear mapping $a_{\theta }$ is an image encoder using a ResNet50 convolutional neural network, followed by a two-layer MLP with ReLU activations as the projection head. The resulting embedding vector from the encoder is fed into a shallow non-linear network, producing the feature vector F . The unit sphere: $f_{nt}=a_{\theta }\left(x_{nt}\right)/\|a_{\theta }\left(x_{nt}\right){\|}_{2}$ projected the feature. F is fed into a single linear layer with as many neurons as there are prototypes. This is the prototype layer C . These prototypes can be regarded as clusters. This layer transforms F into K trainable prototype vectors, and the resulting output is the dot product of F and the prototypes. The “weights” matrix of this layer, updated during backpropagation, can be envisioned as a learnable prototype bank. From this feature, a code $o_{nt}$ is derived by mapping $f_{nt}$ to trainable prototype vectors set $\left\{c_{1},\ldots ,c_{K}\right\}$ . The matrix C represents these columns as $c_{1},\ldots ,c_{K}$ . Finally, a portion of the linear layer’s output is utilized for cluster assignment through the Sinkhorn-Knopp algorithm (Cuturi, 2013), and a swapped prediction problem is established. The output of the Sinkhorn-Knopp algorithm is denoted as O .

The process for generating the codes, as well as updating the prototypes online will now be elaborated. Equation 1 loss function consists of multiple terms. The terms are, predicting $o_{t}$ from $f_{s}$ and $o_{s}$ from $f_{t}$ . These terms have contributed to the “swapped” prediction problem. The cross-entropy loss between $o_{t}$ and the softmax probability $p_{t}$ is essentially what each term represents. The probability was calculated by applying the softmax function to the dot products of $f_{i}$ and all prototypes C ., i.e.,

$$l\left(f_{t},o_{s}\right)=-\underset{k}{\sum }o_{s}^{\left(k\right)}\log p_{t}^{\left(k\right)},\text{ where, }p_{t}^{\left(k\right)}=\frac{\exp\left(\frac{1}{\tau }f_{t}^{T}c_{k}\right)}{\sum_{k^{\prime }}\exp\left(\frac{1}{\tau }f_{t}^{T}c_{k^{\prime }}\right)}$$ (2)

In Equation 2, the temperature parameter is denoted by τ . For the swapped prediction task, the loss function in Equation 3 is obtained by taking the loss from Equation 2 across the pairs of augmentations i.e., $x_{nt}$ , $x_{ns}$ for all the images. Starting with $x_{nt}$ , we obtain $f_{nt}$ , which is then used to calculate $o_{nt}$ in the following equation. A similar process applies to $x_{ns}$ , yielding $f_{ns}$ and subsequently $o_{ns}$ .

$$\begin{array}{l} \frac{1}{N}\sum_{n=1}^{N}\sum_{s,t∼T}\left[\frac{1}{\tau }\,f_{nt}^{T}\,Co_{ns}+\frac{1}{\tau }\,f_{ns}^{T}\,Co_{nt}-\log\sum_{k=1}^{K}\exp\left(\frac{f_{nt}^{T}c_{k}}{\tau }\right)\right.\\ \left.-\log\sum_{k=1}^{K}\exp\left(\frac{f_{ns}^{T}c_{k}}{\tau }\right)\right]\; \end{array}$$ (3)

In Equation 3, loss function is simultaneously reduced concerning C and the parameter values θ of $a_{\theta }$ image encoder $a_{\theta }$ utilized to generate ${\left(f_{nt}\right)}_{n,t}$ features. $f_{ns}$ represents the feature, and $o_{ns}$ is the corresponding code of one augmented view of the n -th image. Similarly, $f_{nt}$ represents the feature, and $o_{nt}$ is the corresponding code of another augmented view of the same n -th image. The number of prototype vectors is denoted by K .

To make this approach online, the codes are computed utilizing just image attributes from a batch. SwAV clusters many instances to the prototypes since they are utilized in various batches. The prototypes C are used to construct codes that partition all of the samples in a batch evenly. This equipartition constraint guarantees that each code inside a batch of images is unique, prohibiting the naive solution in which all images have the same code. The objective is to map a set of B feature vectors $F=\left[f_{1},\ldots ,f_{B}\right]$ to $C=\left[c_{1},\ldots ,c_{K}\right]$ . The mappings are denoted by $O=\left[o_{1},\ldots ,o_{B}\right]$ , and the optimization is focused on maximizing the likeness between the prototypes and the features, as follows:

O*=maxO∈OTrOTCTF+ϵHO, where, HO=−∑ijOij⁡logOij (4)

In Equation 4, the entropy function is denoted by H , and the smoothness parameter is denoted by ϵ . The smoothness parameter is used for the mapping purpose. Utilizing a powerful entropy regularization (increased ϵ for example,) results in a straightforward solution. This is where all samples tumble into a distinct representation and stand evenly distributed among each prototype. Therefore, the ϵ value is kept low. Asano et al. (2019) restricts the matrix O to lie inside the transportation polytope to guarantee an equal partition. The researchers worked on the entire dataset. By restricting the transportation polytope for every minibatch, our model adapted their approach to operate on mini-batches:

$$O=\left\{O\in R_{+}^{K\times B}|O1_{B^{T}}=\frac{1}{K}1_{K},O^{T}1_{K^{T}}=\frac{1}{B}1_{B}\right\}$$ (5)

In Equation 5, the vector of ones in dimension K is represented by $1_{K}$ . These restrictions ensure every prototype is selected within the batch a minimum of $\frac{B}{K}$ times.

To maximize the degree of resemblance between the prototypes and image features, the optimization of C is performed using the Sinkhorn-Knopp algorithm (Cuturi, 2013), which enforces an equipartition constraint. This constraint guarantees that different image features are not mapped to the same prototype. To ensure continuity in the online process, the optimized O is preserved in its continuous form.

After determining a continuous solution $O^{*}$ to Problem 4, Asano et al. (2019) demonstrate a rounding approach to produce a discrete code. Empirical evidence reveals that discrete codes outperform when computed offline on the complete dataset. However, in an online scenario when only minibatches are used, the usage of discrete codes produces lower outcomes than continuous codes. This is owing to the severe optimization necessary for rounding to get discrete codes, which have a higher intensity than gradient updates. Although it promotes speedy model convergence, it frequently results in an inferior solution. As a result, the soft code $O^{*}$ is maintained rather than being rounded. The soft codes $O^{*}$ depict the solution to the problem presented in Equation 4, constrained within the set O , and expressed as a normalized exponential matrix (Cuturi, 2013):

$$O^{*}=\text{Diag}\left(u\right)\exp\left(\frac{C^{T}F}{\epsilon }\right)\text{ Diag}\left(v\right),$$ (6)

In Equation 6, renormalization vectors in $R^{K}$ and $R^{B}$ are denoted as u and v , respectively. The iterative Sinkhorn-Knopp algorithm is used to construct the renormalization vectors with only a few matrix multiplications (Cuturi, 2013).

## 5 Results and discussion

Since training traditional deep learning models works well for a substantial quantity of labeled data, this limits their usefulness in sectors where large annotations are difficult to get. This study validates the proposed contrasting cluster-based SwAV model framework by demonstrating promising classification performance on a small dataset [APTOS 2019 Asia Pacific Tele-Ophthalmology Society (2019)].

SwAV obtained 87.00% classification accuracy on the APTOS 2019 dataset after fine-tuning the hyperparameters (see Table 2). CNN-based EfficientNet-B5, Transformer-based Swin, and ViT obtained accuracy of 83.79%, 57.83%, and 64.15% on APTOS 2019 data, respectively. SwAV converged marginally faster than the other three experimental models, taking only 33 epochs. It takes 35 epochs for both EfficientNet-B5 and Swin. ViT took 50 epochs. Hence, Contrastive Cluster-based SwAV demonstrates a significant performance gain over CNN and Transformer-based models with fewer epochs. The AdamW optimizer was used for all model training in this study. AdamW is an updated variant of the Adam optimizer, which is particularly effective when dealing with large-scale problems involving numerous data points or parameters. The Adam optimizer is known for its efficiency and low memory usage, which aligns well with the SwAV model’s need for low memory consumption as an online learning model. It improves learning by adjusting learning rates based on both the mean of the first-order moment (the gradient) and the mean of the second-order moment (the squared gradient). However, Adam can sometimes be sensitive to initial learning rates and other hyperparameters, potentially affecting the optimizer’s stability and convergence. Additionally, overfitting can be an issue, especially with small datasets. AdamW addresses these limitations, offering better performance and stability. The remaining hyperparameters were selected through fine-tuning. This process involved manually testing various combinations of hyperparameter values to evaluate and optimize the model’s performance during training. Table 2 presents the final optimal hyperparameters for the models trained on the Aptos 2019 dataset.

**TABLE 2:** Optimal hyperparameters of different models for APTOS dataset.

Hyperparameters	SwAV	EfficientNet-B5	Swin	ViT
Optimizer	AdamW	AdamW	AdamW	AdamW
Batch size	64	16	32	64
Learning rate	1e−3	1e−3	1e−5	1e−5
Weight decay	5e−2	5e−2	5e−2	5e−2
Exponential moving avg	0.99	0.99996	0.99	0.99
Epochs	33	35	35	50

In the ablation study of this work, various voting ensemble combinations of CNN and Transformer-based models were also explored alongside Contrastive Clustering-based SwAV. As shown in Table 3, the results illustrate the accuracy of these experiments. Interestingly, even when combining two CNN-based models, EfficientNet-B5, along with Transformer-based architectures like Swin and Vision Transformer (ViT), the performance still falls short of SwAV’s. Nevertheless, SwAV achieved the highest accuracy, showcasing superior precision, sensitivity, and F1-score values.

**TABLE 3:** Ablation study result of different models for APTOS 2019 data.

Method	Accuracy	Precision	Sensitivity	F1-score
Swin	57.83	55.00	58.00	57.00
ViT + SwAV	60.00	60.00	60.00	60.00
ViT	64.15	64.00	64.00	64.00
EfficientNet-B5 + Swin	67.79	62.00	67.00	65.00
Swin + ViT + SwAV	68.01	68.86	68.16	68.00
Swin + SwAV	69.60	66.00	65.70	66.00
EfficientNet-B5 + Swin + ViT	70.54	70.00	70.00	70.00
EfficientNet-B5 + ViT	77.09	73.00	74.00	74.00
EfficientNet-B5 + Swin + ViT + SwAV	80.59	80.00	80.24	80.00
EfficientNet-B5	83.79	83.09	84.00	83.30
EfficientNet-B5 + ViT + SwAV	84.50	84.00	84.00	84.00
SwAV	87.00	86.00	87.00	86.00

**FIGURE 8:**
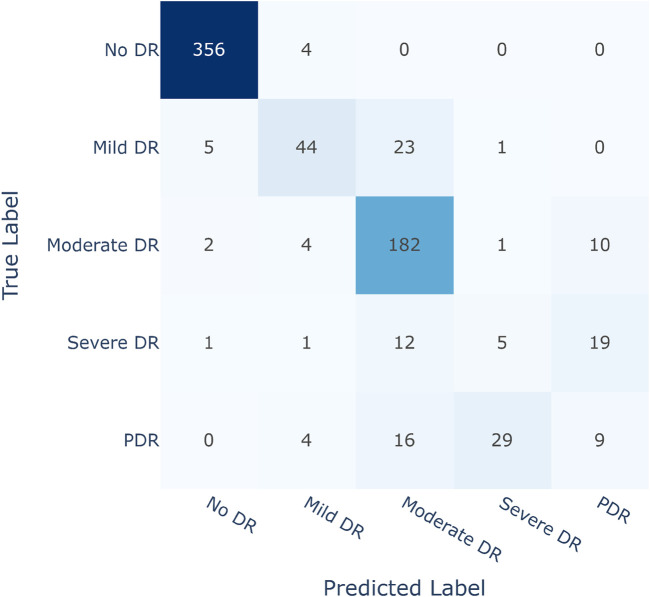
Confusion matrix of SwAV.

Improving sensitivity in medical science research is crucial for correctly identifying affected patients. The main objective of this study is to classify diabetic retinopathy according to severity levels and encourage people to seek medical assistance before their condition worsens. As a result, it is critical to avoid misidentifying any of the four Diabetic Retinopathy (DR) conditions as Non-Diabetic Retinopathy (No DR) or healthy. The suggested DR grading architecture, employing the Contrastive Cluster-based SwAV algorithm, efficiently achieved this purpose, as indicated by the validation in the confusion matrix depicted in Figure 8.

The APTOS 2019 dataset was used by a number of studies to assess DR severity. The classification accuracy of state-of-the-art models using this dataset is listed down in Table 4. The studies did not provide class-wise results. Yang et al. (2024) attained 85.6% accuracy using a novel transformer-based model TMILv4. However, one limitation of their work is low sensitivity (73.7%), which is critical in medical science-based research. In the work of Shaik and Cherukuri (2021), Convolutional autoencoder and neural support vector machine (LA-NSVM) are combined and trained and acquired a performance accuracy of 84.31%. However, the work still lacks in sensitivity score (66.16%). Kassani et al. (2019) achieved a relatively higher sensitivity (88.24%) using a Modified Xception network even though their work has a lower overall accuracy (83.09%). The remaining studies in Table 4 exhibit comparatively lower performance in terms of either accuracy or sensitivity. SwAV beats all these state-of-the-art CNN and Transformer-based models in terms of weighted F1-score, accuracy, precision, and sensitivity with a score of 86%, 86%, 87%, and 87% respectively (Table 4).

**TABLE 4:** Classification performance compared to CNN and Transformer-based state-of-the-art models for APTOS 2019 dataset.

Method	Accuracy	Precision	Sensitivity	F1-score
CapsNet + VGG16 (Kumar et al., 2021)	75.50	—	—	—
NASNet (Dondeti et al., 2020)	77.90	76.00	77.00	
MobileNet (Kassani et al., 2019)	79.01	—	76.47	—
DNN + Blended VGG and Xception (Bodapati et al., 2020)	80.96	—	—	—
Inception-ResNet-v2 (Gangwar and Ravi, 2021)	82.18	—	—	—
Composite Gated attention DNN (Bodapati et al., 2021a)	82.54	82.00	83.00	82.00
Modified Xception (Kassani et al., 2019)	83.09	—	88.24	—
Modified VGG16 (Bodapati et al., 2021b)	84.31	—	—	84.00
LA-NSVM (Shaik and Cherukuri, 2021)	84.31	75.86	66.16	69.90
TMILv4 (Yang et al., 2024)	85.60	—	73.70	—
SwAV	87.00	86.00	87.00	86.00

**FIGURE 9:**
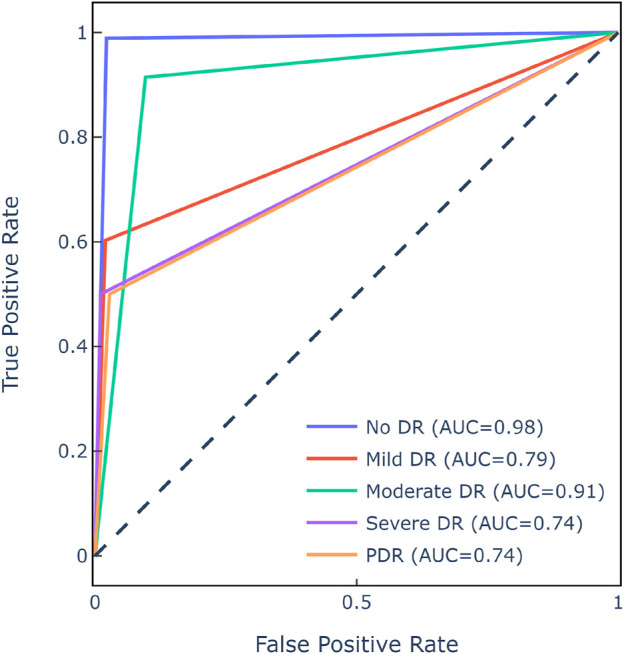
ROC matrix of SwAV for APTOS 2019 dataset.

Figure 9 displays the class-wise ROC, which evaluates the SwAV’s ability to discern between DR levels. The SwAV model had an estimated ROC of 83.2% for the APTOS 2019 dataset. Despite an imbalanced dataset, the model’s resilience was demonstrated by decent ROC values for each class.

According to Table 5, the majority of state-of-the-art models extracted features and identified DR from fundus images using transfer learning techniques. The transfer learning models, such as VGG16 with 138.3 million parameters and ResNet50 with 50 layers, are overly complex. The models also needed high-resolution retinal fundus images to accurately distinguish DR levels. The other models given in Table 5 also have a large number of parameters and layers, which takes too long to train and converge. In comparison, SwAV has just 16 layers and 23.5 million parameters. Based on the comparison, the suggested SwAV can reliably classify DR levels with fewer parameters, layers, and faster processing times. The model can accurately classify fundus images in under 2 μ s, enabling real-time patient feedback.

**TABLE 5:** Simplicity of proposed approach in comparison with state-of-the-art models.

Model	No. of layers	No. of parameters (in million)
NasNet-Large (Liu et al., 2020; Bodapati et al., 2020)	—	88.9
EfficientNetB5 (Liu et al., 2020)	—	30.5
Inception Resnet-v2 (Liu et al., 2020; Bodapati et al., 2020)	164	55.8
ResNet50 (Qummar et al., 2019; Kassani et al., 2019)	50	25.6
Inception-v3 (Qummar et al., 2019; Kassani et al., 2019)	48	23.8
VGG16 (Bodapati et al., 2020; 2021a)	16	138.3
Proposed SwAV	16	23.5

## 6 Conclusion

This study introduces a novel method for self-supervised multiclass grading of Diabetic Retinopathy by employing a Contrastive Cluster-based SwAV model. Initially, retinal fundus images underwent cropping to eliminate spherical z-space, followed by Gaussian filtering to address uneven brightness and enhance lesion visibility. Augmentations were subsequently applied before normalization. The processed images were then fed into SwAV with a ResNet50 backbone for DR severity classification. By integrating adept pre-processing techniques and incorporating Contrastive Cluster Assignments of retinal images with the SwAV classifier, the system’s prediction accuracies are significantly enhanced compared to traditional deep learning approaches. SwAV attained an accuracy of 87%, surpassing state-of-the-art CNN and Transformer-based models despite its simpler architecture comprising 16 layers and 23.5 million parameters. The ablation study further demonstrated SwAV’s superiority over ensemble methods involving CNN and Transformer-based models. This efficient computer-based approach to DR grading reduces reliance on costly routine medical checkups. Given the absence of symptoms in the early stages of Diabetic Retinopathy for most cases, undiagnosed individuals can initiate medical diagnosis using this framework. In the future, the focus will be on seamlessly incorporating this method into everyday clinical practice. Overall, the proposed approach shows great potential for making a substantial contribution to the prevention and control of DR, improving the condition of life for millions of people worldwide.

## Data Availability

Publicly available datasets were analyzed in this study. This data can be found here: https://www.kaggle.com/c/aptos2019-blindness-detection/data.
